# 2.Q. Workshop: Prepared for the unexpected: using foresight to address uncertainty

**DOI:** 10.1093/eurpub/ckac129.119

**Published:** 2022-10-25

**Authors:** Organised by: RIVM (Netherlands), Chair persons: Henk Hilderink (EUPHA-FS), Ana Vieira (Netherlands)

## Abstract

All future trends carry uncertainty in them. For example, the COVID-19 pandemic showed great uncertainty about current and future health impacts and how the virus may directly impact our health. Foresight (as a systematic, participatory, future-intelligence-gathering and medium-to-long term vision-building process aimed at enabling present-day decisions and mobilising joint actions) explicitly addresses uncertainty. Doing a foresight study one has to deal with different sources of uncertainty. Next to commonly known statistical uncertainty, foresight studies have to deal with cognitive uncertainty, i.e. uncertainty related to the limited knowledge that we have regarding the complexity of the underlying system or limited knowledge of what future economic growth will be. A better understanding of the location, level, and nature of the cognitive uncertainties helps assess the robustness of future scenarios. In addition to cognitive uncertainty, normative uncertainty are distinguished. This refers to uncertainty related to differences in what we consider a desirable future. Especially in the field of public health people differ on what they consider to be “good health”. In this workshop, we will focus on the different aspects of uncertainty and how they are considered in existing foresight studies. The workshop will start with an interactive Mentimeter session to better understand how the audience is familiar with foresight and uncertainty. Then, a brief presentation is given as introduction to foresight and a systematic way of accounting for uncertainty, explaining the basic concepts to level understanding of the audience. This is followed by a presentation of a Foresight study (FRESHER) that addresses cognitive uncertainties consistently and systematically. The second foresight study (Dutch PHFS, RVIM) is a good example of how normative uncertainties are considered. Finally, the last presentation (CEG-IST) gives the policy perspective about how policy makers can incorporate uncertainty, as modelled through foresight, into policy evaluation. The workshop will be concluded by a discussion with the three presenters on the lessons learned regarding foresight and uncertainty.

**Key messages:**

• Foresight studies addressing uncertainty are essential to be better prepared for the future.

• Acknowledging different types of uncertainty is needed to support foresight-informed policy making.

